# A viable mouse model of factor X deficiency provides evidence for maternal transfer of factor X

**DOI:** 10.1111/j.1538-7836.2008.02849.x

**Published:** 2008-02

**Authors:** S J TAI, R W HERZOG, P MARGARITIS, V R ARRUDA, K CHU, J A GOLDEN, P A LABOSKY, K A HIGH

**Affiliations:** * Division of Hematology, The Children’s Hospital of PhiladelphiaPhiladelphia, PA; † Department of Pediatrics, University of Pennsylvania School of MedicinePhiladelphia, PA; ‡ Department of Pathology, University of Pennsylvania School of MedicinePhiladelphia, PA; § Department of Cell and Developmental Biology, University of Pennsylvania School of MedicinePhiladelphia, PA; ¶ Howard Hughes Medical Institute, The Children’s Hospital of PhiladelphiaPhiladelphia, PA, USA

**Keywords:** coagulation, development, factor X, mouse model

## Abstract

*Background:*Activated factor X (FXa) is a vitamin K-dependent serine protease that plays a pivotal role in blood coagulation by converting prothrombin to thrombin. There are no reports of humans with complete deficiency of FX, and knockout of murine *F10* is embryonic or perinatal lethal. *Objective:*We sought to generate a viable mouse model of FX deficiency. *Methods:*We used a socket-targeting construct to generate *F10*-knockout mice by eliminating *F10* exon 8 (knockout allele termed *F10*^tm1Ccmt^, abbreviated as ‘−’; wild-type ‘+’), and a plug-targeting construct to generate mice expressing a FX variant with normal antigen levels but low levels of FX activity [4–9% normal in humans carrying the defect, Pro^343^→Ser, termed FX Friuli (mutant allele termed *F10*^tm2Ccmt^, abbreviated as F)]. *Results:F10* knockout mice exhibited embryonic or perinatal lethality. In contrast, homozygous Friuli mice [*F10* (F/F)] had FX activity levels of ∼5.5% (sufficient to rescue both embryonic and perinatal lethality), but developed age-dependent iron deposition and cardiac fibrosis. Interestingly, *F10* (−/F) mice with FX activity levels of 1–3% also showed complete rescue of lethality. Further study of this model provides evidence supporting a role of maternal FX transfer in the embryonic survival. *Conclusions:*We demonstrate that, while complete absence of FX is incompatible with murine survival, minimal FX activity as low as 1–3% is sufficient to rescue the lethal phenotype. This viable low-FX mouse model will facilitate the development of FX-directed therapies as well as investigation of the FX role in embryonic development.

## Introduction

The hemostatic process is a complex, highly regulated and dynamic sequence of events that ultimately results in the localized formation of a stable clot in response to vascular damage [1]. Activated coagulation factor X (FXa; a vitamin K-dependent, two-chain plasma serine protease) catalyzes the conversion of prothrombin to thrombin and occupies a pivotal role in the coagulation cascade. Under physiological conditions, cleavage of FX to form FXa is initiated by the FVIIa/tissue factor (TF) complex and maintained by the FIXa/FVIIIa complex.

Several lines of evidence suggest that FX/FXa may have functions other than hemostasis, such as promoting cytokine release from endothelial cells and initiating signaling via protease activated receptors (PARs) [2,3]. Interestingly, no humans with deletion of both *F10* genes have been reported. Instead, all of the described mutations are either missense mutations or compound heterozygotes with a deletion or stop codon on one allele and a missense mutation on the other, suggesting that complete deficiency of FX is incompatible with life (http://www.hgmd.cf.ac.uk/ac/index.php) [4]. Similar findings have been described in mice, in which targeted disruption of the *F10* gene is associated with loss of ∼50% of homozygous affected animals *in utero*, and loss of the remaining affected mice as a result of bleeding complications within a few days of birth [5].

In this study, we sought to develop a viable mouse model of FX deficiency and to determine whether very low levels of FX activity could rescue the embryonic and perinatal lethality. We used a ‘plug and socket’ targeting strategy; the ‘socket’ targeting construct eliminated exon 8 and its 3′ flanking sequence following homologous recombination, to give rise to *F10*-knockout mice (knockout allele *F10*^tm1Ccmt^ abbreviated as ‘–’; wild-type *F10* allele abbreviated as ‘+’) exhibiting embryonic and perinatal lethality [6]. We chose exon 8 as almost 50% of the reported *F10* mutations are located within this exon. We used a second (plug) targeting construct to reconstitute the *F10* gene with a variant exon 8 containing a Pro^343^→Ser substitution (FX Friuli, allele *F10*^tm2Ccmt^ abbreviated as F), which, in humans homozygous, results in FX activity levels of 4–9% and normal antigen levels [7]. Homozygous FX Friuli mice [*F10* (F/F)], with expression directed by the endogenous *F10* promoter, yielded FX activity levels of 5.5%, and complete rescue of embryonic and perinatal lethality of FX deficiency. We used this genetic approach to further define the role of FX in embryonic survival and generated mice with the *F10* (−/F) genotype. We demonstrate that FX activity levels of 1–3% were sufficient for complete rescue of embryonic and perinatal lethality, and provided evidence for a contribution of maternal transfer of FX activity to embryonic survival. These mice can be used in studies of novel therapies for FX deficiency as well as probing the role of FX in processes such as sepsis and metastasis.

## Methods

### Isolation of murine F10 genomic DNA

Designed from the exon 8 sequence of the murine *F10* gene, a 350-bp fragment from 129/SvJ mouse genomic DNA (Stratagene, La Jolla, CA, USA) was amplified using polymerase chain reaction (PCR) and sequenced. This was used as a probe to screen a bacterial artificial chromosome (BAC) library derived from *Hind*III, partially digested embryonic stem (ES) 129/SvJ DNA (Genome Systems, St Louis, MO, USA). A 7-kb *Nhe*I-digested fragment was isolated from a positive BAC clone containing exons 7 and 8, the intervening intron, and the flanking region downstream of exon 8. This fragment also was used to design and construct the *F10* socket-targeting vector.

### Construction of the F10 socket- and plug-targeting vectors

**Fig. 1:**
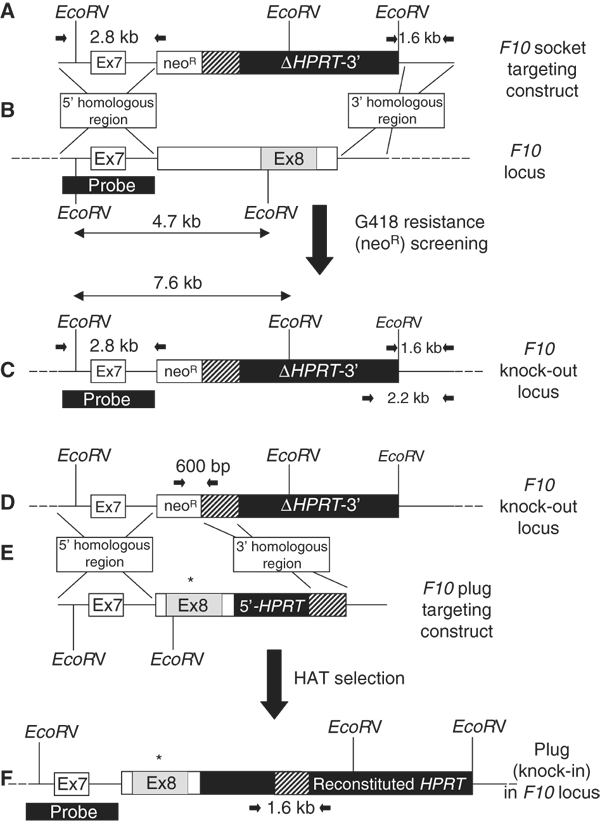
Plug and socket targeting strategy for generation of *F10* knockout and Friuli mice. (A) ‘Socket’ targeting construct consists of 2.8 kb 5′targeting arm and 1.6 kb 3′ targeting arm derived from 3′untranslated region of murine *F10* gene. The targeting arms flank a functional *Neo* gene and a partially deleted *HPRT* gene (Δ*HPRT*). (B) The 3′ end of the murine *F10* locus. (C) After homologous recombination between the socket construct (A) and the murine *F10* locus (B), exon 8 is replaced with the *Neo* gene and partial *HPRT* gene. (D) *F10* knockout locus showing deleted exon 8 replaced by *Neo* and partial *HPRT*. (E) *F10* plug targeting construct contains variant exon 8 with Friuli mutation (*) and the missing 5′ portion of the *HPRT* gene, flanked by 5′ and 3′ targeting arms. (F) Reconstitution of the *HPRT* gene after homologous recombination allows selection of correctly targeted clones on HAT medium. Arrows indicate primer pairs used for polymerase chain reaction identification. The darkened bar shows the location of the probe for Southern blot.

Engineered in the pPD20 plasmid vector (provided by Dr Randy Thresher, UNC-Chapel Hill, USA), the *F10* socket-targeting construct consists of 5′ (2.8-kb including intron 6, exon 7, and part of intron 7 of the murine *F10* gene) and 3′ [1.6-kb containing the sequence downstream of the putative polyadenylation signal (ATTAAA) and the overlapping termination codon (TAA) of the *F10* gene] homologous sequences flanking a functional neomycin (*Neo*) gene and a partially deleted, non-functional *HPRT* minigene (Δ*HPRT*) (Fig. 1A) [6]. The *F10* plug-targeting construct cloned in pBY9 plasmid has the identical 5′arm as the *F10* socket-targeting vector; its 3′arm contains a 1.4-kb overlapping *HPRT* sequence with the pPD20 plasmid (Fig. 1E). In the plug-targeting construct, exon 8 has been modified by site-directed mutagenesis at the codon for amino acid 343 (in mouse FX protein) to generate the Pro^343^→Ser (FX Friuli) mutation (CCC→TCC) [7].

### Electroporation, selection, analysis of ES cell clones and DNA extraction

*HPRT*-deficient murine ES cells (E14TG2a) were grown as described [8] and electroporated with 100 μg of *Sac*II-linearized *F10* socket-targeting vector and G418-selected. For the *F10* plug-targeting construct, *HPRT*-deficient murine ES cells heterozygous for deletion of exon 8 (from the original targeting event) were electroporated as above and selected based on G418 sensitivity and growth in HAT medium [9]. ES cell clone DNA as well as liver DNA from neonates was isolated using the Easy DNA Kit (Invitrogen, Carlsbad, CA, USA). All ES cell clones were identified by PCR and/or Southern blot. For adult mice, genomic DNA was extracted from blood samples using the QIAamp DNA Blood Mini Kit (Qiagen, Valencia, CA, USA).

### Generation of F10 (−/−), F10 (F/F), and F10 (−/F) mice

The production of all chimeric mice was performed by the Transgenic & Chimeric Mouse Facility of the University of Pennsylvania (USA). Correctly targeted *F10* (+/−) and *F10* (+/F) murine ES cell clones were expanded, micro-injected into C57BL/6 blastocysts, and implanted into the uterine horns of pseudo-pregnant mice. The chimeric males were mated with wild-type C57BL/6 females to generate heterozygous *F10* (+/−) or *F10* (+/F) offspring, which were intercrossed to obtain homozygous *F10* (−/−) or *F10* (F/F) mice, respectively. *F10* (−/F) mice were generated by crossing homozygous *F10* (F/F) females with heterozygous *F10* (+/−) males.

### Reverse transcription-polymerase chain reaction and Northern blotting of total RNA isolated from liver tissues

Total RNA was isolated from liver of *F10* (+/+) and *F10* (−/−) newborn mice, using TRIzol reagent (Invitrogen), reverse transcribed into first-strand cDNA using oligo(dT) primers (Invitrogen) and PCR-amplified for exons 1–6 and exon 8. For Northern blot analysis, total RNA (20 μg) was isolated from liver of neonates, and hybridized with a *F10* cDNA fragment corresponding to exons 1–6. A β-actin cDNA probe was used as a control. Band intensity was determined by densitometry.

### Western blot analysis on plasma samples and protein samples isolated from liver tissues

Plasma samples or liver protein extracts were obtained from *F10* (+/+), *F10* (+/−), and *F10* (−/−) neonates and from *F10* (+/+), *F10* (+/−), and *F10* (F/F) adult mice and analyzed by Western blotting [10]. A horseradish-peroxidase (HRP) conjugated rabbit antihuman FX polyclonal antibody (1:300 dilution; Cedarlane, ON, Canada) or an affinity-purified sheep antihuman FX polyclonal antibody (1:250 dilution; Cedarlane), followed by an HRP-conjugated rabbit antisheep immunoglobulin (1:1000 dilution; Dako, Carpinteria, CA, USA) were used.

### Activated partial thromboplastin time and prothrombin time FX assays

Citrated plasma collected from neonates and adult mice (4–6 weeks old) was used to measure FX coagulation activities by activated partial thromboplastin time (aPTT) and prothrombin time (PT) assays (diluted 1:40 for aPTT, 1:80 for PT), as previously described [11]. Clotting times were converted to percent normal FX activity using a pooled normal mouse plasma standard curve.

### Histologic analysis of FX-deficient mice

Adult mice were sacrificed by CO_2_ inhalation; organs were harvested and fixed in 10% formalin overnight at 4 °C. Tissues were embedded in paraffin, sectioned (5 μm per section), and stained with hematoxylin and eosin, Prussian blue, or Masson’s Trichrome stain.

## Results

### Construction of mice with targeted disruption of exon 8

With the expectation that a deletion of murine *F10* would result in lethality, which would then require rescue to produce a viable FX-deficient mouse model, we chose to use the ‘plug-and-socket’ targeting strategy originally described by Detloff *et al.* [6]. As well as being different from the previously described FX deficiency mouse model [5], this approach has the advantage that it allowed us to inactivate and then replace the targeted region with another sequence of interest, which would then express a low-activity FX variant under the control of the endogenous promoter and its regulatory sequences. Using a targeting construct (socket) designed to delete exon 8 (Fig. 1A), we electroporated *HPRT*-deficient murine ES cells and identified correctly targeted ES clones (8/192 screened, 4%) that were expanded and micro-injected into C57BL/6 blastocysts. The resulting chimeric males were mated with wild-type C57BL/6 females. Subsequently, heterozygous *F10* (+/−) mice were intercrossed to obtain homozygous *F10* (−/−) offspring.

### Characterization of FX-deficient (−/−) neonates

**Fig. 2:**
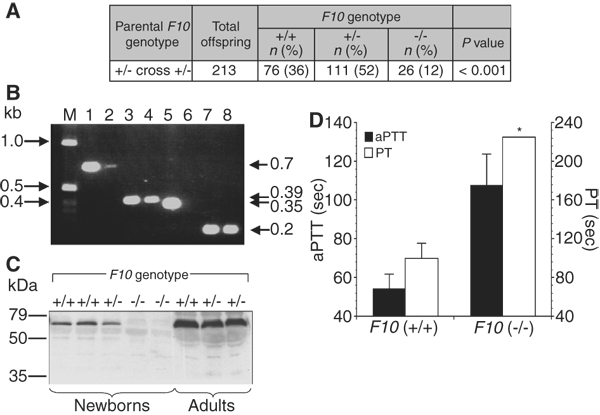
Characterization of *F10* (−/−) mice. (A) Genotype distribution among offspring of heterozygous *F10* (+/−) intercross. *F10* (−/−) mice are under-represented. *n* = number of animals; *P* values are compared to normal Mendelian distribution (25%, 50%, 25%) using a chi-squared test. (B) Reverse transcription-polymerase chain reaction analysis for *F10* transcripts on RNA isolated from newborn mouse liver. M, molecular weight marker. Odd numbered lanes, *F10* (+/+) mice; even numbered lanes, *F10* (−/−) mice; lanes 1–2, exons 1–6; lanes 3–4, exons 5–7; lanes 5–6, exon 8; lanes 7–8, β-actin cDNA. (C) Western blot analysis on mouse plasma from *F10* (+/+), (+/−), and (−/−) newborn mice, or from *F10* (+/+) or *F10* (+/−) adults [note that *F10* (−/−) mice do not survive to adulthood]. The 55 kD signal from mouse factor X is readily detected in *F10* (+/+) and *F10* (+/−) animals but not in *F10* (−/−) newborn mice. (D) Coagulation times (s) on newborn mice. Times are averages for 6–31 mice per genotype. Asterisk denotes clotting time >200 s.

DNA analysis of the full-term offspring (*n* = 213) resulting from mating of heterozygous *F10* (+/−) mice showed that *F10* (−/−) newborns (12%) were under-represented (*P* < 0.001; Fig. 2A), confirming the occurrence of partial embryonic lethality and exhibiting similar pathology and survival (data not shown) as previously described [5]. Analysis of *F10*-null embryos showed that as early as embryonic day 11.5, *F10* (−/−) embryos accounted for only 14% of progeny (*n* = 104, *P* < 0.05), indicating that most of the *F10* (−/−) embryo loss had occurred by mid-gestation.

Further analysis of the *F10* (*−*/*−*) mice showed absence of a correctly sized *F10* transcript by Northern blot on total liver RNA, although a very faint, larger than wild-type *F10* transcript was observed (data not shown), most likely containing part of the *Neo*-Δ*HPRT* cassette. More sensitive methodology (reverse transcription-PCR) revealed a markedly reduced, but nonetheless detectable, signal for exons 1–6 (Fig. 2B, lane 2) and, as expected, absence of signal for exon 8 (Fig. 2B, lane 6). The faint, larger than wild-type *F10* transcript, most likely accounted for the amplification of exons 1–6 as well as 5–7 (Fig. 2B, lane 4). Corroborating these results, *F10* (*−*/*−*) mice did not have any detectable circulating FX antigen by Western blot [in contrast to their wild-type and *F10* (+/*−*) littermates; Fig. 2C] and, as expected, exhibited prolonged clotting times (Fig. 2D).

### Knock-in of F10 Friuli and characterization of mice homozygous for variant FX

**Fig. 3:**
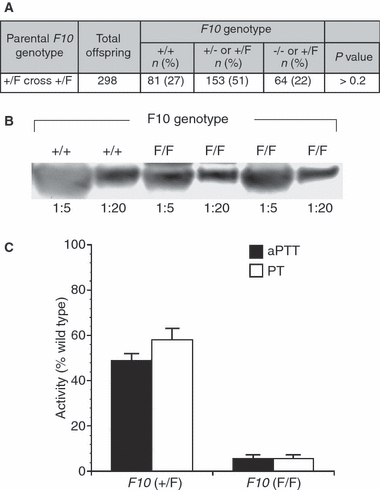
Characterization of factor X Friuli [*F10* (F/F)] mice. (A) Genotype of live offspring of heterozygous crosses. Mice homozygous for the Friuli knock-in are born at the expected frequency [22%, compared to *F10* (−/−) mice born at 12% from a *F10* (+/−) intercross]. *n* = number of animals; *P* values are compared to normal Mendelian distribution (25%, 50%, 25%) using a chi-squared test. (B) Western blot analysis of plasma from adult mice [*F10* (+/+) or *F10* (F/F)] at two dilutions (as indicated). Antigen levels are similar for both genotypes. (C) Coagulation activity of adult mice (*n* = 4 per genotype) from *F10* (+/F) intercross (expressed in % wild-type).

Clearly, the embryonic and perinatal mortality of the *F10* (−/−) mice limit their utility for studies of FX therapeutics. We therefore sought to rescue the mortality by restoring a low level of FX expression under the control of the endogenous *F10* promoter. We selected an exon 8 mutation from the human mutation database that is associated with reduced but not absent FX activity, FX Friuli. This missense mutation (Pro^343^→Ser) in the catalytic domain exhibits normal antigen but reduced activity levels of 4–9% [7]. Given the extensive sequence and functional conservation in the catalytic domain of FX across species, we were able to generate a plug-targeting construct containing the identical mouse *F10* gene mutation, expected to have a similar functional effect. We carried out a retargeting experiment on the original, targeted murine ES cells to generate mice expressing the FX Friuli mutation. The use of two selectable markers results in a high rate of correctly retargeted ES cells; 9/48 clones examined (19%), in contrast to 8/192 (4%) from the original targeting event, were correctly targeted as assessed by PCR and Southern blot analyses. The results of the F1 intercross of *F10* (+/F) mice are summarized in Fig. 3A [compared to results of the F1 intercross of *F10* (+/−) mice in Fig. 2A]. These show that *F10* (F/F) mice are born at the expected frequency based on Mendelian inheritance, confirming that the Friuli variant rescues embryonic lethality. Secondly, perinatal lethality is also rescued; indeed, survival is comparable to that of littermates with the *F10* (+/F) genotype (80% at 18 months). Determination of FX antigen and activity levels in the *F10* (F/F) mice reveals normal antigen levels as judged by Western blot (Fig. 3B), and intrinsic and extrinsic activity of 5.5 ± 1.8% and 5.5 ± 1.9%, respectively (Fig. 3C). As expected, *F10* (+/F) mice exhibited approximately 50% intrinsic/extrinsic activity (Fig. 3C). Thus, the murine model of FX Friuli is similar to the human mutation in terms of phenotype, activity, and antigen levels. Furthermore, the reproductive fitness of *F10* (F/F) mice is indistinguishable from that of the wild-type mice; female *F10* (F/F) mice have litter sizes similar to those of wild-type females (data not shown).

### Survival of mice with very low FX levels [F10 (−/F)]

The homozygous Friuli mice [*F10* (F/F)], although useful as a model for FX deficiency, nevertheless failed to define a lower limit of FX activity required for survival. To generate and characterize mice with even lower levels of FX, we crossed the *F10* (F/F) females with *F10* (+/−) males to generate *F10* (−/F) offspring [*F10* (F/F) females rather than *F10* (+/−) females were used in this mating scheme to confirm the reproductive competence of these mice]. Such matings showed the expected genotypic distribution (Table 1, *P* > 0.2). Intrinsic and extrinsic FX activity in the *F10* (−/F) mice was 1.1 ± 0.1% and 2.9 ± 0.2%, respectively (Table 1). Postnatal survival of the *F10* (−/F) is normal, clearly demonstrating that FX activity levels as low as 1–3% are adequate to rescue embryonic and perinatal mortality in *F10* (−/−) mice. One hundred percent of *F10* (−/F) mice survived for the first postnatal month and ∼80% have survived for at least 15 months.

**Table 1:** Characterization of *F10* knockout/Friuli [*F10* (−/F)] heterozygotes

		*F10* genotype		
Parental *F10* genotype	Total offspring	+/F *n* (%)	−/F *n* (%)	*P* value *
F/F (♀) cross +/− (♂)	97	52 (54%)	45 (46%)	>0.2
aPTT (Sec [%]) Normal *F10* (+/+) range: 35.5 ± 1.6 (100%)			74.1 ± 1.3 (1.1%) *n* = 4	
PT (Sec [%]) Normal *F10* (+/+) range: 34.5 ± 2.1 (100%)			97.3 ± 2 (2.9%) *n* = 4	

* Values are compared to normal Mendelian distribution (25%, 50%, 25%) using chi-squared test. aPTT, activated partial thromboplastin time; PT, prothrombin time.

### Histologic analysis of FX-deficient mice

**Fig. 4:**
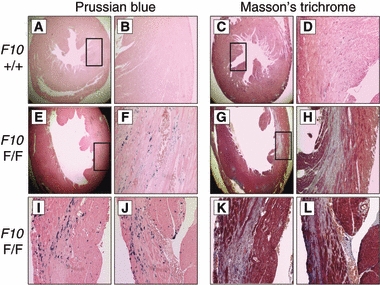
Histology of heart tissue from wild-type and *F10*-Friuli [*F10* (F/F)] mice sacrificed at 10 months of age. Prussian blue and Masson’s Trichrome stains on *F10* (+/+) mice (A)–(D) and *F10* (F/F) mice (E)–(L). Prussian blue stain is positive for iron deposition in *F10* (F/F) mice but not in wild-type mice. Similarly, Masson’s Trichrome stain is positive for fibrosis in *F10* (F/F) mice but not in wild-type mice. (A), (C), (E), (G) Magnification 10×; (B), (D), (F), (H) magnification 40×; (I), (J), (K), (L) magnification 100×.

The *F10* (F/F) and *F10* (–/F) mice demonstrated relatively normal survival through the postnatal period. However, a previous report documents that both low TF (∼1%) and low FVII (∼1%) mice exhibit cardiac fibrosis shown to have resulted from recurrent episodes of hemorrhage into the myocardium from cardiac vessels [12]. We therefore examined cardiac tissue from 10-month-old *F10* (F/F) mice, and observed similar findings – iron deposition in the myocardium (Prussian blue staining) and co-localized fibrosis (Masson’s Trichrome staining; Fig. 4E–L) – in contrast to age-matched littermates (Fig. 4A–D). These findings are consistent with the observations made by Pawlinski *et al.* [12] in low TF and low FVII mice. Examination of *F10* (F/F) mice at an earlier time point (three months of age) failed to disclose evidence of iron deposition or cardiac fibrosis (data not shown). These results suggest that both age and genotype of the mice are important contributors to development of cardiac fibrosis in these FX-deficient mice.

### Role of maternal FX in embryonic survival

Viable and reproductively fit, the Friuli mice [*F10* (*−*/F)] provided an opportunity to assess the role of maternal FX levels in embryonic survival, as it enabled a comparison of *F10* (*−*/F) intercross offspring (maternal FX activity levels ∼1–3%, antigen levels ∼50%) with *F10* (+/*−*) intercross offspring (maternal FX antigen and activity levels ∼50%). The data (Table 2) demonstrate that there is a statistically significant decrease in *F10* (*−*/*−*) offspring (2% of total progeny) in the *F10* (*−*/F) intercross compared to *F10* (*−*/*−*) offspring (12% of total progeny) in the *F10* (+/*−*) intercross (*P* = 0.0045, see Fig. 2A), suggesting that maternal transfer of FX occurs during development and that low FX activity levels in the maternal circulation result in reduced survival of *F10* (*−*/*−*) embryos.

**Table 2:** The effect of maternal factor X activity* levels on embryonic survival

		*F10* genotype			
Parental *F10* genotype	Total offspring	F/F *n* (%)	−/F *n* (%)	−/−^‡^*n* (%)	*P*-value^†^
−/F cross −/F	89	29 (33%)	58 (65%)	2 (2%)	<0.001

* All parental genotypes exhibit 50% antigen levels. †Values are compared to normal Mendelian distribution (25%, 50%, 25%) using chi-squared test. ^‡^*P* value = 0.0045 of *F10* (+/−) intercross compared to *F10* (−/F) intercross in Fig. 2A (Fisher’s exact test).

## Discussion

Severe FX deficiency has a low incidence, of the order of one case per 500 000, and complete absence of FX has never been reported. A recently compiled database (http://www.hgmd.cf.ac.uk/ac/index.php) [4] confirms this finding. These data in humans are consistent with the data presented here; mice with homozygous deletion of the *F10* gene show a reduction in numbers occurring as early as ∼E11.5 and are thus born in reduced numbers (approximately 50% of expected numbers), exhibiting 100% perinatal mortality.

Because of an interest in developing new approaches for the treatment of bleeding disorders [13–16], and to analyze the potential role of FX in pathophysiological processes such as sepsis and metastasis [17,18], we wished to develop a viable murine model of severe FX deficiency. The only extant model [15] requires *in utero* transplantation of wild-type fetal liver, a cumbersome manipulation and not transmissible from one generation to the next. The plug-and-socket methodology utilized here facilitates repeated modification of a specific locus as well as expression of the gene of interest under the control of the endogenous promoter, thus preserving the temporal and spatial patterns of expression during embryonic development. Exon 8 was chosen because we wanted to target a region of the *F10* gene that encodes variants with a range of activities, as nearly half of the reported patients with FX deficiency have mutations in exon 8.

We restored FX expression using a FX variant with extremely low activity and normal antigen levels, FX Friuli [7]. Based on extensive conservation of the catalytic domain between species, we hypothesized that (i) the mutant murine protein would have activity levels similar to that of the human protein, and (ii) these levels would be adequate to rescue both embryonic and postnatal lethality. Both of our hypotheses proved to be correct; the FX Friuli [*F10* (F/F)] mice have normal antigen levels, and activity levels quite similar to those of homozygous affected humans (5.5% in mice, 4–9% in humans), and these levels are adequate to rescue both embryonic and postnatal lethality. From *F10* (−/F) mice, we also established that FX activity levels of only 1–3%, and/or antigen levels of ∼50%, are adequate for such rescue. At the molecular level, the murine Friuli mutation in exon 8 most likely causes a similar structural perturbation in the catalytic domain as has been proposed for its human counterpart [19], thus affecting its catalytic potential. Clearly, further studies will help to define the role of this mutation in the mechanistic and molecular interactions of FX within the coagulation cascade.

The Friuli mice, viable but with low circulating levels of FX, can be used to address the role of maternal clotting factor levels on embryonic survival, a point of uncertainty in the literature. Although our data are indirect, a comparison of two intercrosses [*F10* (−/F) intercross and *F10* (+/−) intercross; Table 2] strongly suggests that embryonic survival of FX-deficient mice is improved by the presence of biologically active FX in the mother. For example, *F10* (−/F) and *F10* (+/−) mothers had distinct levels of FX activity (1–3% and ∼50%, respectively) but similar antigen levels (∼50%), suggesting that presence of maternal FX antigen is not sufficient to improve embryonic survival. Thus, the partial penetrance of the embryonic lethality in *F10* null embryos is, at least partly, a function of maternal transfer of FX. A probable biological significance of maternal transfer of FX (and other coagulation factors in general) may be the prolongation of survival of embryos with FX or other coagulation deficiencies that would otherwise succumb. Moreover, although relative embryonic survival of *F10* knockout mice [*F10* (−/−)] is directly dependent on the specific genotype of the mother (with respect to FX activity levels), our data suggest that embryonic and postnatal survival of *F10* (−/F) mice is independent of the mother’s genotype. This is clearly shown in both the *F10* (F/F) × *F10* (+/−) cross and the *F10* (−/F) intercross (Tables 1 and 2, respectively), where *F10* (−F) mice were born in numbers that establish lack of embryonic lethality.

In summary, we have shown that both embryonic and postnatal lethality of FX deficiency can be rescued by ‘knock-in’ of a variant FX molecule with reduced activity and normal antigen levels (FX Friuli), and that this mouse model accurately models the mild human bleeding diathesis. Moreover, *F10* (−/F) mice had FX antigen levels of ∼50% and activity levels of 1–3%, and also survived the embryonic and postnatal periods. Our strategy has made it possible to separate the roles of antigen and activity in embryonic development. It represents a genetic approach to assessing the role of maternal transfer of FX biological activity in embryonic survival, and demonstrates that mere presence of FX antigen is not sufficient. Additionally, these mice provide a viable model for those interested in studying novel therapies for FX deficiency, for investigation of the causes of embryonic lethality in FX-deficient mice and for the role of FX in biological processes such as sepsis and metastasis.
